# Restoring private autism dataset from sanitized database using an optimized key produced from enhanced combined PSO-GWO framework

**DOI:** 10.1038/s41598-024-66603-y

**Published:** 2024-07-09

**Authors:** Md. Mokhlesur Rahman, Ravie Chandren Muniyandi, Shahnorbanun Sahran, Opeyemi Lateef Usman, Md. Moniruzzaman

**Affiliations:** 1 Centre for Cyber Security, Faculty of Information Science and Technology, Universiti Kebangsaan Malaysia, 43600 UKM Bangi, Selangor Malaysia; 2 Centre for Artificial Intelligence Technology, Faculty of Information Science and Technology, Universiti Kebangsaan Malaysia, 43600 UKM Bangi, Selangor Malaysia; 3 Department of Electrical and Electronic Engineering, College of Engineering and Technology, International University of Business Agriculture and Technology, Uttara, Dhaka, 1230 Bangladesh; 4 Department of Computer Science, Tai Solarin University of Education, P.M.B. 2118, Ijagun, Ogun State Nigeria

**Keywords:** Data restoration, Optimal key, Security and privacy, Autism dataset, PSO-GWO framework, Information technology, Computer science

## Abstract

The timely identification of autism spectrum disorder (ASD) in children is imperative to prevent potential challenges as they grow. When sharing data related to autism for an accurate diagnosis, safeguarding its security and privacy is a paramount concern to fend off unauthorized access, modification, or theft during transmission. Researchers have devised diverse security and privacy models or frameworks, most of which often leverage proprietary algorithms or adapt existing ones to address data leakage. However, conventional anonymization methods, although effective in the sanitization process, proved inadequate for the restoration process. Furthermore, despite numerous scholarly contributions aimed at refining the restoration process, the accuracy of restoration remains notably deficient. Based on the problems identified above, this paper presents a novel approach to data restoration for sanitized sensitive autism datasets with improved performance. In the prior study, we constructed an optimal key for the sanitization process utilizing the proposed Enhanced Combined PSO-GWO framework. This key was implemented to conceal sensitive autism data in the database, thus avoiding information leakage. In this research, the same key was employed during the data restoration process to enhance the accuracy of the original data recovery. Therefore, the study enhanced the restoration process for ASD data's security and privacy by utilizing an optimal key produced via the Enhanced Combined PSO-GWO framework. When compared to existing meta-heuristic algorithms, the simulation results from the autism data restoration experiments demonstrated highly competitive accuracies with 99.90%, 99.60%, 99.50%, 99.25%, and 99.70%, respectively. Among the four types of datasets used, this method outperforms other existing methods on the 30-month autism children dataset, mostly.

## Introduction

Autism Spectrum Disorder (ASD) is a developmental disorder that is revealed in early childhood, and it includes an impairment of social communication and interactions along with restricted and repetitive behaviour. An increasing number of children diagnosed with ASD is occurring worldwide^1,2^. Children with ASD have difficulties with social communication, social interaction and show restricted and repetitive behaviour. The Diagnostic and Statistical Manual of Mental Disorders (DSM-5)^3^, in its fifth edition, has categorized children with ASD into three distinct levels, depending on the extent of supports required to carry out daily activities: requiring support, requiring substantial support, and requiring very substantial support. Moreover, children diagnosed with ASD encounter secondary difficulties; for example, the inability to communicate verbally (non-verbal), an intellectual disabilities such as dyslexia and attention deficit hyperactivity disorder (ADHD), suffer from depression, and experience epilepsy, etc.

Till date, the causes and cure for autism remain unclear. Therefore, a number of studies have demonstrated the importance of early diagnosis and evidence-based treatments, which may significantly improve the quality of life for individuals with ASD. Despite the fact that many studies are being conducted to classify children with ASD using advanced methods^4^, researchers are emphasizing the need for autism data security and privacy^5^ in this regard. For the reasons stated above, the security and privacy of an increasing amount of autism data should be a major concern in the early diagnosis of ASD, as these data can be leaked through network data transmission. Data protection is now maintained by researchers in a variety of ways. Researchers are attempting to reduce the increasing disruption of data flow or the threat of hacking the data by utilizing a variety of advanced techniques^6–11^. Some studies are emphasizing data sanitization process using optimized key, whereas only a few studies have proposed a privacy-preserving restoration procedure for autism data with limited performance accuracies^12–23^. Data restoration refers to the retrieval of backup data from secondary storage and its reinstatement to its initial or an alternative location^24^. The procedure is crucial in guaranteeing the effective recovery of genuine data and promoting computer network development. In the context of electronic healthcare data storage system belonging to children with ASD, data restoration is particularly significant to safeguard data integrity and preclude unauthorized access or modifications. However, the information gathered is saved as personal health records at a hospital or medical centre. Personal health records are now stored in electronic form to ensure access by all members and are referred to as ASD electronic health records regarding autism data^25^. These electronic records are used to collect and store various types of data each member collects^26^. Furthermore, these electronic records enable data sharing among doctors, therapists, computer scientists, teachers, social workers, and family members. This electronic data sharing prioritises privacy and security, particularly to avoid data misuse and violation^27–29^. Therefore, the researchers have developed a number of security and privacy frameworks^30–34^; some of which are based on the privacy-preservation paradigm and cutting-edge technologies to protect datasets^35–38^.

However, to solve the security and privacy concerns, a few of these studies make use of conventional meta-heuristic techniques^12,14^. Yet, the local searching ability, convergence speed, and solution precision of standard meta-heuristic algorithms are all worse. Additionally, several of these studies make advantage of query and k-anonymity^23^. Such methods require a significant investment of time and computing power. Moreover, the authors in^14^ formed optimal key, but there were some issues in forming the key, and the fitness function was not appropriate. Yet again, a framework was proposed in the study of^16^, where the authors created appropriate sanitization key and enhanced the local searching ability, convergence speed, etc., but they never used this key in the restoration process, which would be a promising finding regarding data restoration. Similarly, the authors in^17,18^ proposed respective techniques where a sanitization key was created, which was not appropriate; thus, the fitness function was inappropriate, and their outcomes had limitations.

Therefore, this research determined certain important problems stated above in the previous study^12,14,17,18,24^, which it resolved and turned into the main subject of this research contributions to the field. These crucial concerns comprise, but are not restricted to, the following:

i. Which value will determine how long the key is?
ii. Which value is the key range?
iii. How long will it take for the key value to be updated throughout the key generation step, which is utilized in the data restoration process?
iv. How will the parameter values be defined?
v. What will be the data restoration protocols?

In order to overcome the aforementioned problems, the study focuses on the data restoration process by utilizing the appropriate sanitization key for security and privacy regarding autism sensitive data. Data restoration is a crying need for safeguarding vulnerable sensitive information. However, the contributions and objectives of this present study are thus, summarized below:

i. To improve the restoration performance by utilizing an optimized key and considering the security and privacy concerns.
ii. To implement the data sanitization method initially proposed in^16^ and thereafter, perform restoration process without compromising the confidentiality and privacy of ASD datasets.
iii. Finally, to compare the performance of the proposed technique with the existing methods.

The structure of the remaining work unfolds as follows: “Related works” explores different encryption and decryption methods which form the basis of the literature review. “Methodology” explains the proposed approach. “System configuration and simulations” presents the experimental results, findings, and analyses surrounding ASD datasets obtained from the implementation of the proposed method, whereas “Discussions” reveals summarized discussions, implications of the proposed method, and limitations as well. Finally, in “Conclusion”, the article provides a concluding remark as well as future research directions.

## Related works

In this section, the work presents a review of related studies on the security and privacy of sensitive data, their characteristics, methods or techniques, and open challenges that must be addressed with specific focus on restoration.

A reseaerch work developed a novel Anonymous Authentication (AA) protocol for the Internet of Medical Things (IoMT) that utilized light-weight Elliptic Curve Cryptography^9^. Their work addressed security issues that were identified as a significant vulnerability in a previous AA scheme proposed by another research work^10^. Another study conducted an analysis of the security measures, technologies, and management frameworks related to cloud computing, specifically, in the healthcare domain^11^. The study examined a range of methods to safeguard medical data and identified both challenges and potential solutions using both established and novel techniques. Additionally, it proposed several models that could be employed to address the identified issues in order to achieve optimal outcomes.

A privacy-preserving data mining technique was introduced^12^, where authors enhanced an algorithm, namely the Opposition Intensity-based Cuckoo Search Algorithm (CSA), by modifying the Cuckoo Search Algorithm. In their proposed technique, they formed an appropriate key for data restoration, but there was no consideration of the mechanism of key management protocols, so this is an issue of considering the key management techniques that may hamper the proper restoration of sensitive data. Additionally, a method for distributed clustering was introduced that involved the transfer of sanitized data to a cloud service provider by a helper user in the study of^13^. The effectiveness of this approach was evaluated based on three metrics: transmission time, processing time, and clustering accuracy. Yet, again, a model was proposed, which utilized Artificial Bee Colony (ABC) optimization algorithm to sanitize sensitive information in their proposed model^14^. Another sanitization method called Improved Maximum Sensitive Itemsets Conflict First (IMSICF) algorithm was proposed for privacy-preserving utility mining (PPUM)^15^. In this method, maximum conflicts in victim itemsets from sensitive itemsets are tallied to be concealed. This approach chooses transactions with a smaller number of non-sensitive itemsets and a high utility of concealed sensitive itemsets for modification in order to minimize the side effects on non-sensitive information.

The authors in^16^ utilized the two optimization algorithms to form a framework called an enhanced combined PSO-GWO meta-heuristic algorithm framework. This framework generated an optimal key for hiding sensitive ASD datasets as an improved method of sanitization process. There was no consideration of data restoration. The authors suggested the need for restoring sensitive data for the security and privacy of medical data after improving their proposed data sanitization process. They mentioned that the same key was promising to restore the sensitive data. The study of^17^ discussed the procedures for yielding an optimal key that was employed in the sanitization process as well as the restoration process, but there were shortcomings for taking a longer time to update the key and no fixation of the number of key ranges. The same concern should be addressed for the study of^18,19^, where the outputs were lacking in optimization^18^ and ineffectiveness to preserve other sensitive information, such as frequent items^19^. Another approach, namely the Bee-Foraging Learning-based Particle Swarm Optimization (BFL-PSO) algorithm, was introduced to yield the optimal key for data sanitization and restoration^20^. The study considered multi-objective functions, such as error rate, computational time, complexity, etc., to measure its performances, but the performances should be more promising. The same authors in^21^ proposed a secured algorithm where they utilized Harmonic Encryption (HE) to fend off attacks for the privacy of sensitive data regarding different diseases like heart disease, diabetes, and cancer. Nonetheless, it needed protocols for time management regarding the analysis of encryption time and decryption time. A further research was conducted on the privacy of electrical health records in the cloud^22^. Mainly, the author developed an approach, namely Elephant Herding Optimization with Opposition-based Learning (EHO-OBL), where they applied blockchain technology to ensure authentication for data integrity. However, the key generation time by the EHO-OBL approach might be enhanced, which was used for data integrity. Moreover, the K-anonymity algorithm was utilized in another study to secure health data privacy^23^. The shortcomings of this approach are that it relies on specific healthcare data, and its reliability or performance is not optimized as well.

Finally, Table 1 provides a brief overview of various recent studies that have implemented advanced techniques or algorithms for data privacy, along with their identified characteristics and challenges.

**Table 1:** An overview of methods, characteristics and challenges of different models for data privacy.

Years/references	Methods/algorithms	Characteristics/attributes	Open challenges/research issues
2019^12^	Opposition Intensity-based CSA algorithm PPDM technique	Hiding sensitive data Using optimal key	Requires significantly longer time to update the key Number of key ranges Mechanisms of key management need to be considered No consideration on combination with web mining
2019^13^	ABC algorithm	Users are grouped by k-means clustering algorithm A helper user from each group performs the data transmitting task Analysing performance based on a few factors such as processing time, clustering accuracy, and data transmission time	Only the kernel k-means algorithm cannot encrypt large datasets in a distributed system
2020^14^	ABC algorithm	Anonymising sensitive data Forming optimal key	In terms of privacy preservation, time and space dimensions were not taken into account
2020^15^	IMSICF algorithm	Consider maximal utility count in sensitive itemsets and minimal number of non-sensitive itemsets	Other types of sensitive information, for instances, frequent and utility itemset preservation
2021^16^	Enhanced Combined PSO-GWO	Anonymising sensitive data using optimal key Enhanced Combined PSO-GWO yields optimal key	Restoration of sensitive data
2021^17^	WNU algorithm	Hiding information Creating optimal key	Needs considerably longer time to update the key Number of key ranges
2022^18^	J-SSO algorithm JA & SSO algorithms	Sensitive data preservation	Yet to be achieved optimized outputs
2022^19^	R-GDA algorithm DA algorithm GWO algorithm	Data security and privacy	Ineffectiveness to preserve other sensitive information, such as frequent item
2023^20^	BFL-PSO algorithms	Medical data security	Required optimized outputs
2023^21^	CCRBM-WOA algorithm IoT sensor devices Homomorphic encryption (HE) model	Various medical data security	Essential time management for encryption & decryption
2024^22^	EHO-OBL technique	Data privacy & integrity	Promising performances required
2024^23^	K–anonymity	Healthcare data privacy	Lack of optimization for high reliability

*CSA* Cuckoo Search Algorithm, *PPDM* Privacy-preserving data mining, *ABC* Artificial Bee Colony, *IMSICF* Improved Maximum Sensitive Itemsets Conflict First, *PSO-GWO* Particle Swarm Optimization-Grey Wolf Optimization, *WNU* Whale with New Crosspoint‐based Update, *J-SSO* Jaya-based Shark Smell Optimization, *JA* Jaya Algorithm, *SSO* Shark Smell Optimization, *R-GDA* random-based grey dragon algorithm, *DA* Dragonfly Algorithm, *GWO* Grey Wolf Optimization Algorithm. *BFL-PSO* Bee-Foraging Learning-based Particle Swarm Optimization, *CCRBM-WO* Centered Convolutional Restricted Boltzmann Machines-based Whale Optimization, *IoT* Internet of Things, *HE* Homomorphic Encryption, *EHO-OBL* Elephant Herding Optimization with Opposition-based Learning.

## Methodology

The purpose of this investigation was to identify a viable fix or solution for a problem. In light of this, the issue that needed to be solved was how the restoration process worked appropriately and how to generate optimal keys utilizing the features of meta-heuristic algorithms. To find the best solution, the study analyzed a number of state-of-the-art approaches to the issues. As such, this investigation has determined a study gap concerning the restoration process and the optimal key formation in those cutting-edge solutions. The introduction part highlighted some noteworthy concerns about security and privacy, for which there is yet no clear technological answer in those cutting-edge technologies. Thus, by correctly creating the optimal key and restoration process, the research was able to address these important concerns.

In order to provide the clarification of the issue, this section talks over the main architecture of the proposed framework and its entire working procedures regarding data restoration.

### Main architecture

**Figure 1:**
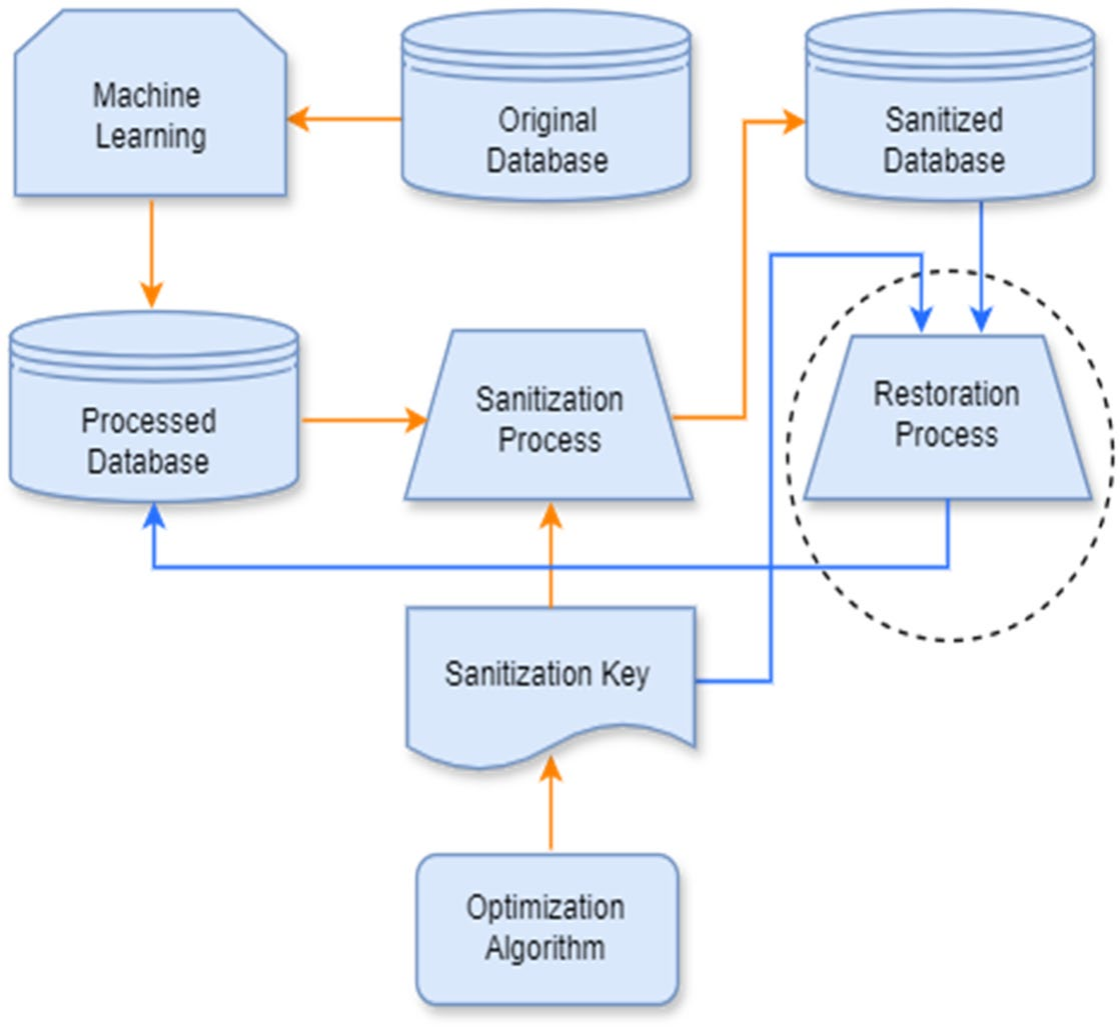
Overall architecture for data security and privacy model^16^.

The proposed approach's general architecture is depicted in Fig. 1, with the goal of maintaining high prediction accuracy while protecting the confidentiality and privacy of autism data. The main components of the recommended framework, for instance, are the autism dataset as original data, support vector machine (SVM), processed database, sanitization process, restoration process, sanitization key, PSO, and GWO algorithms. The original database is a raw database that is not prepared to be utilized, because some values can be missing, irregular, irrelevant, or null. So, the original data needs to be pre-processed. Support Vector Machine is employed on the original datasets in order to get the processed database. Using SVM, the datasets have been transformed into an understandable format. It manages the missing and duplicate values. It presents all the information, such as the total number of attributes, instances, data types, number of missing values, maximum and minimum values, error values, etc., easily and quickly. If any anomaly occurs in the datasets, it can be solved easily. Now, the processed database is ready to use because it has already been transformed into an understandable and desired format with relevant data, not null or missing values. By getting the processed datasets, it may be sanitized through the sanitization process by providing sanitization key produced by the enhanced combined PSO-GWO approach. There are two optimization algorithms, such as particle swarm optimization (PSO) and grey wolf optimization (GWO), which are employed in this recommended framework. In the framework, the characteristics of PSO have been incorporated into GWO to enhance the capability of convergence as well as local searching ability, whose main purpose is to yield the best key for data sanitization and data restoration. The sanitization process is a process where processed data is hidden by the presence of the sanitization key. Different techniques, such as reconstruction of the key matrix, the Khatri-Rao process, binarization, the XOR operation, and so on, have been applied in the process. However, a sanitized database is one that is secured, protected. This sanitized database can also be utilized to archive the original data in the presence of a sanitization key through restoration process. The restoration process is a technique where an authorised person can get the processed data again in the presence of the sanitized database and sanitization key. The restoration phase is denoted by the blue arrow, while the sanitization phase is denoted by the dark orange arrow.

Sensitive autism data is preserved using data restoration process. In this paper, the authors analyse the restoration process depicted in the broken circle and put emphasis on the issue of autism data privacy. From the overall architecture, the restoration process consists of the following components:

1. Processed database
2. Sanitization key
3. Sanitized database
4. Restoration process, and
5. Optimization algorithms.

**Figure 2:**
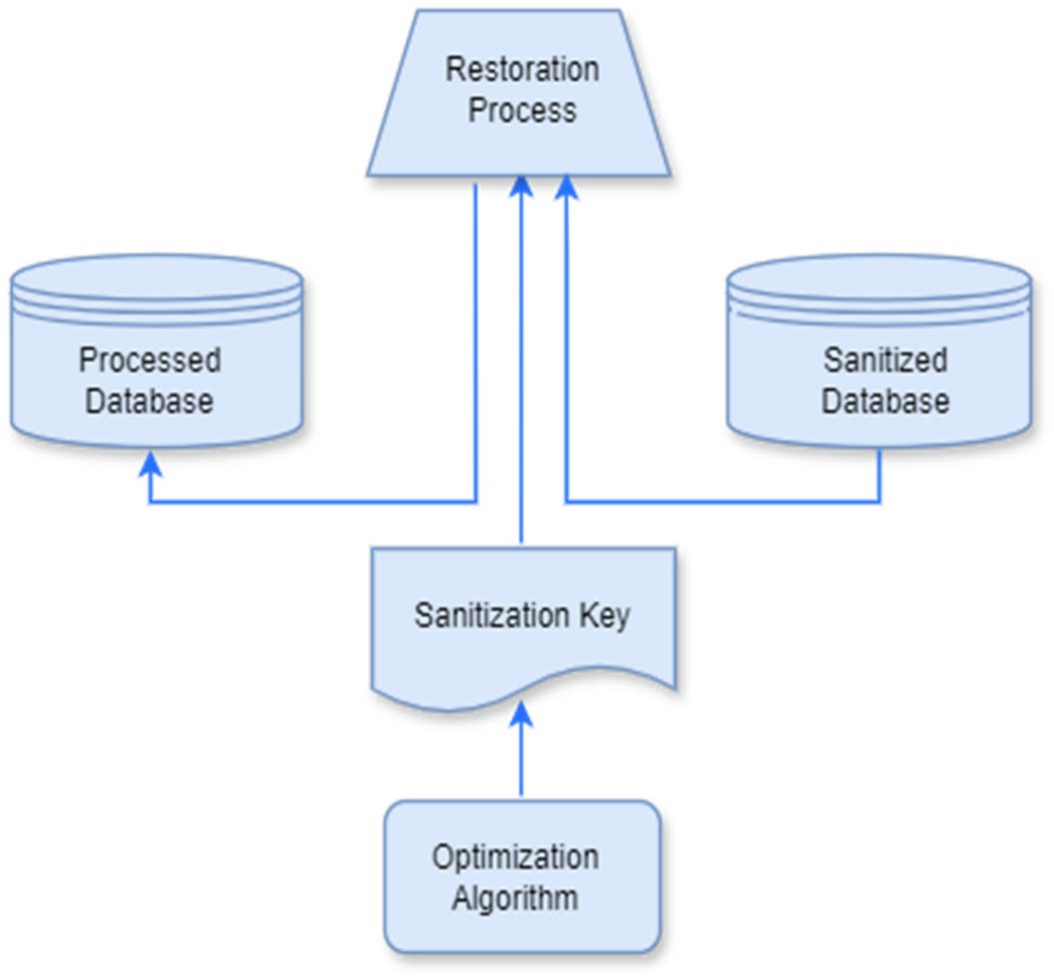
Architecture of data restoration process.

The interactions among the components of the restoration process are depicted in Fig. 2. The restoration process, including the decoding process and a restoration algorithm, is illustrated in the following subsections broadly.

The symbols used in this section are illustrated as below:

**Table:** 

Symbols	Descriptions
*D*	Processed (from original) database
*D* ${\prime}$	Sanitization database
*K*_1_,* K*_2_….*K*_*N*_	Number of keys
*K*_2_	Pruned key matrix
⊕	XOR operator
+	Binary summation
*S*^*m*^	Randomly moved space
*S*	Prior moved space
*C*_1_, *C*_2_, *C*_3_	Objective functions
*f*_s_	Frequency of sensitive itemset in sanitized data
*f*_*m*_	Frequency of sensitive itemset in original data
*f*_*ns*_	Frequency of non-sensitive itemset in sanitized data
*w*1,* w*2,* w*3	Impact of a particular cost function
*f*	Fitness function
*G*	Minimum objective function
$\overrightarrow{M}$	Location of the particle
$\overrightarrow{w}$	Velocity of the particle
*ω*	User-defined behavioral parameter (an inertia weight)
$\overrightarrow{q}$	Particle’s previous best position (pbest position)
$\overrightarrow{f}$	Particle’s previous best position in the swarm (gbest position)
*r*_1_*, r*_2_	Stochastic variables
*c*_1_*, c*_2_	Acceleration constants
*u*	Current iteration
$\overrightarrow{H}$, $\overrightarrow{E}$	Coefficient vectors

### Data restoration

**Figure 3:**
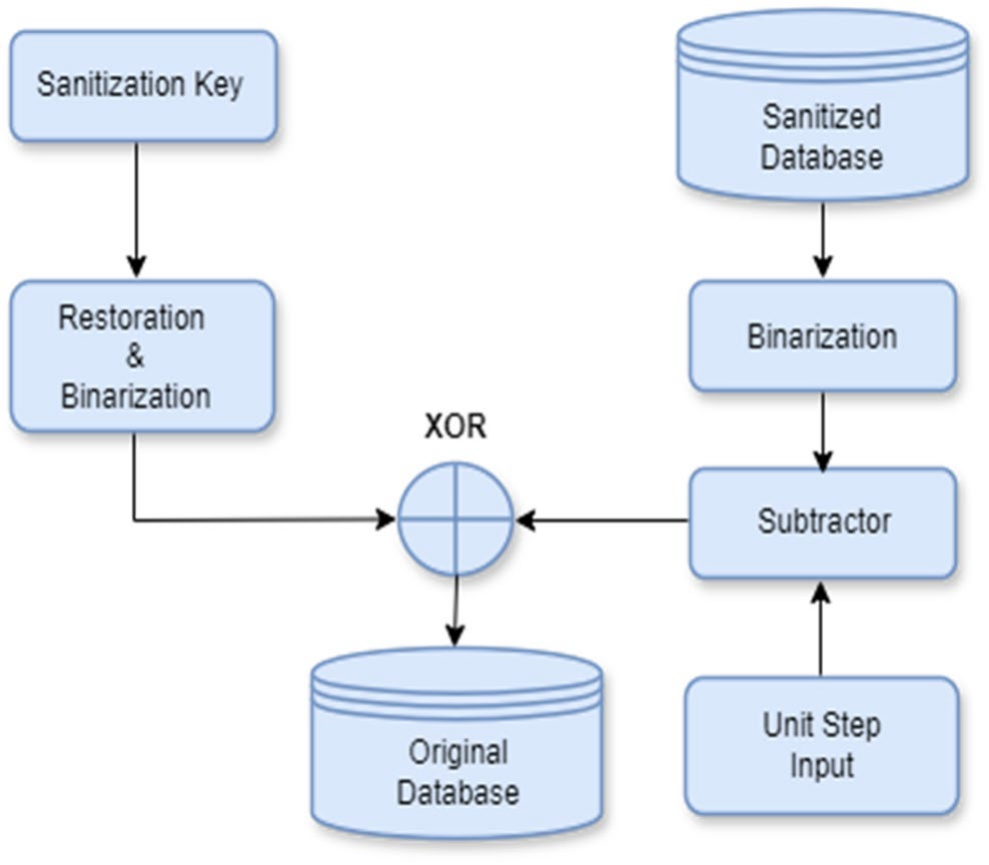
Architecture of decoding process.

Figure 3 depicts the decoding procedure for data restoration. From the sanitization process and key generation process, the sanitized database, *D’* and pruned key matrix, *K*_2_ are obtained that are revealed in Eq. (1)^16^,

1 $$D {\prime}={(K}_{2} \oplus D )+1,$$

However, in this decoding procedure, *D*′ and *K*_2_ must be binarized. From binarization block, the sanitized database is reduced by considering unit value of input step size. In the interim, the XOR task is performed on the minimized sanitization database and the binarized key matrix, and consequently the restored database is recaptured.

Furthermore, it is noted earlier that key generation procedure yields sanitized key, which is employed to restore database *D*. This sanitized key is used to generate sanitization database *D’* from where restored database is achieved by using Eq. (2) below:

2 $$\widehat{D}=\left({D}{\prime}-1\right)\oplus {K}_{2},$$

where $\widehat{D}$ implies restored data and *K*_2_ is the sanitizing key matrix generated from *K*. Algorithm 1 demonstrates the pseudo code of the whole restoration procedure as below:

**Algorithm 1:**
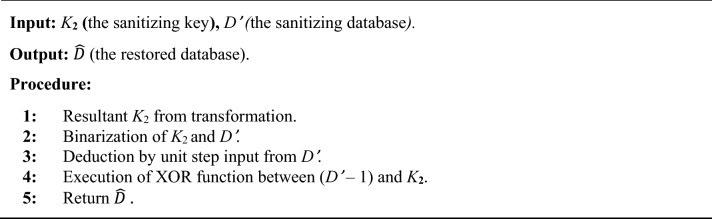
Pseudo code for restoration process.

To sum up, from the restoration process algorithm, the *D’* attained from the sanitization procedure and *K*_**2**_ from the key generation procedure must be binarized. The binarized database from the binarization procedure is deducted from the unit step input. In the interim, the database being subtracted and the binarized key matrix executes a xor operation, and thus the restored database, $\widehat{D}$, is produced.

### Evaluation of objective functions

The three objective functions, such as *C*_1_, *C*_2_, as well as *C*_3_ (*C*_1_ refers to the hiding failure rate, *C*_2_ represents the information preservation rate, and *C*_3_ denotes the degree of modification), are evaluated by Eq. (3) through Eq. (6)^16^. Following the generation of association rules from original database and sanitized database, as well as sensitive rules, this evaluation is carried out.

3 $${C}_{1}= \frac{fs}{fm},$$

4 $${C}_{2}= \frac{fns}{fm},$$

5 $$ {C_3} = dist \, ( \, D,D^{\prime}),\;\;\;\;\;\;dist \to Euclidian \, distance $$

6 $$f =w1\left(\frac{\text{C}1}{{max}\left[\text{C}1, \text{C}2\right]}\right)+ w2\left(1-\frac{\text{C}2}{{max}\left[\text{C}1, \text{C}2\right]} \right)+w3 \left(\frac{\text{C}3}{{max}(\text{C}4)}\right),$$

wherein, *f*_s_ indicates the frequency of sensitive items regarding sanitized data, *f*_*m*_ implies the frequency of sensitive items in the matter of original data, *f*_*ns*_ denotes the non-sensitive items frequency on the subject of sanitized data, *C*_3_ is the Euclidean distance, additionally, the original data, *D*, and the sanitised data, *D’*, together yield the *C*_3_, *C*_4_ represents the distance between each item in the collection of sanitised and original data, *w*_1_, *w*_2_, *w*_3_ show a certain cost function affecting *C*_1_, *C*_2,_ and *C*_3_ simultaneously, eventually, *f* specifies the fitness function of the suggested technique.

However, the objective functions *C*_1_, *C*_2,_ and *C*_3_ are chosen to find out how well the autism data is disinfected by applying the suggested Enhanced Combined PSO-GWO framework. Consequently, the objective functions of the recommended approach are expressed by utilizing the following Eq. (7) for the medical datasets as below^16^:

7 $$ G = Min\left( f \right),\;\;\;\;\;\;\;Min \to minimum$$

### Working procedure of the proposed framework with PSO & GWO optimization algorithms

The three vectors in the Particle Swarm Optimisation (PSO) algorithm are called *x*-, *p*-, and *v*-vectors. *x*-vector tracks the particle's current position in the search area, *p*-vector (pbest) indicates the position where the particle has found the best solution as possible, and *v*-vector includes particle velocity, which indicates the future locations of each other particle during the iteration. The first shifting of particles in predetermined directions is done at random. The particle started to migrate in the direction of the previous optimal position on its own since its orientation could be changed gradually. Next, it searches the neighbourhood for the ideal locations to perform certain fitness functions, using the formula, fit = *S*^*m*^* − S*. In this instance, the particle's position is given as $\overrightarrow{M}$*∈ S*^*m*^, while its velocity is specified as $\overrightarrow{w}$. These two variables are first chosen at random and thereafter updated often in accordance with the two formulas, which are displayed in the following Eqs. (8) and (9) as below^16^:

8 $$\overrightarrow{w}=\omega \overrightarrow{w}+{c}_{1}{r}_{1}\left(\overrightarrow{q}-\overrightarrow{M} \right)+{c}_{2}{r}_{2}\left( \overrightarrow{f}-\overrightarrow{M}\right)$$

9 $$\overrightarrow{M}\leftarrow \overrightarrow{M}+\overrightarrow{w}$$

Here, an inertia weight, ω, is an example of a user-defined behavioural parameter that controls the amount of particle velocity recurrence. Both particle's prior best position (pbest position) and its prior best position within the swarm (gbest position) are specified as $\overrightarrow{q}$ and $\overrightarrow{f}$, respectively. The particles interact with one another implicitly in this way. Furthermore, *r*_1_, *r*_2_ ∼ *U* (0, 1) are used as stochastic variables to weight this, and *c*_2_, *c*_1_ stand for acceleration constants. Velocity is introduced to the present location so that particle can move towards next location in the search region, irrespective of fitness upgrades that is depicted above in Eq. (9).

Whereas, there exists a hierarchy of search agents, for instance, level 1 (Alpha), level 2 (Beta), level 3 (Delta), as well as level 4 (Omega) for the Grey Wolf Optimization (GWO) technique. At the time of hunting their prey, the criteria for encircling the prey are stated mathematically through the Eq. (10) to Eq. (11) as follows^16^:

10 $$\overrightarrow{B}=\left| \overrightarrow{E}.{\overrightarrow{M}}_{q}\left(u\right)-\overrightarrow{M}\left(u\right)\right|$$

11 $$\overrightarrow{M}\left(u+1\right)={\overrightarrow{M}}_{q}\left(u\right)-\overrightarrow{H}. \overrightarrow{B}$$

At this point, the current iteration is denoted by *u* as well as the coefficient vectors are represented by $\overrightarrow{E}$,$\overrightarrow{H}$. Moreover, the heightened understanding of likely prey sites possessed by alpha, beta, and delta wolves is utilized to computationally replicate the hunting behaviours of grey wolves. No matter whether the remaining amount is needed, the initial best three solutions are considered. The mathematical modelling of the best three solutions is provided in Eqs. (12), (13), and (14), respectively^16^.

$${\overrightarrow{B}}_{\alpha }=\left| {\overrightarrow{E}}_{1}.{\overrightarrow{M}}_{\alpha }-\overrightarrow{M}\right|$$

$${\overrightarrow{B}}_{\beta }=\left| {\overrightarrow{E}}_{2}. {\overrightarrow{M}}_{\beta }-\overrightarrow{M}\right|$$

12 $${\overrightarrow{B}}_{\delta }=\left| {\overrightarrow{E}}_{3}\cdot {\overrightarrow{M}}_{\delta }-\overrightarrow{M}\right|$$

$${\overrightarrow{M}}_{1}= {\overrightarrow{M}}_{\alpha }- {\overrightarrow{H}}_{1}. ({\overrightarrow{B}}_{\alpha })$$

$${\overrightarrow{M}}_{2}= {\overrightarrow{M}}_{\beta }- {\overrightarrow{H}}_{2}. ({\overrightarrow{B}}_{\beta })$$

13 $${\overrightarrow{M}}_{3}= {\overrightarrow{M}}_{\delta }- {\overrightarrow{H}}_{3}. ({\overrightarrow{B}}_{\delta })$$

14 $$\overrightarrow{M}\left(u+1\right)= \frac{{\overrightarrow{M}}_{1} + {\overrightarrow{M}}_{2} + {\overrightarrow{M}}_{3}}{3}$$

Although conventional algorithms have certain drawbacks, such as reduced performance across multiple domains in the case of the traditional PSO algorithm and limited local search capability, slower convergence, and lower solution accuracy for the GWO algorithm, there is room for improvement. To enhance their effectiveness and integration, further investigation is necessary. As an attempt, this study implements a novel hybrid algorithm to solve these issues highlighted above. In the anticipated Enhanced Combined PSO-GWO, the characteristics of the PSO algorithm are employed into the GWO optimization procedure. The Eqs. (10) and (11) present mathematical model for the prey enclosure in the proposed approach, while Eqs. (12), (13), and (14) illustrate the mathematical model for the hunting method. The primary reformation in the proposed paradigm is the update of the location. Thus, making update for the location in Enhanced Combined PSO-GWO framework is represented in Eq. (15), wherein $\overrightarrow{M}$ denotes the velocity to update the location of PSO as revealed in Eqs. (8) to (9).

15 $$M\left(u+1\right)= \frac{{\overrightarrow{M}}_{1} + {\overrightarrow{M}}_{2} + {\overrightarrow{M}}_{3} + \overrightarrow{M}}{4}$$

Furthermore, the acceleration constants *c*_1_ and *c*_2_ in the conventional PSO regarded as constants, but in the suggested Enhanced Combined PSO-GWO framework, *c*_1_, *c*_2_ change in accordance with the values 0.1, 0.3, 0.5, 0.7, and 1.0.

## System configuration and simulations

This section explains how the proposed technique for restoring autism data works. The various types of datasets with source are discussed in "Dataset description". "Simulation setup" reveals the simulation setup, whereas in "Results and discussions", the paper also demonstrated how the proposed technique performed in simulations when the acceleration constants, *c*_1_ and *c*_2_, were varied.

### Dataset description

The obtained datasets regarding autism from Faculty of Education at Universiti Kebangsaan Malaysia are utilized for this study^39^. The datasets utilized in the experiments encompassed autistic children of various ages, such as dataset of autistic children at 24 months comprising of 209 instances along with 26 attributes, at 30 months 209 instances and 29 attributes, at 36 months 234 instances and 31 attributes, and at 48 months featuring 302 instances, 33 attributes. All the datasets were categorized as diagnostic data of autism, with scoring choices: *z* = 0, *v* = 5, and *x* = 10. The cut-off values for each dataset differed, with values of 71, 95, 100, and 105, respectively. Datasets were validated by 8 experts in early childhood development measuring behaviour, self-control, compliance, communication, self-adjustment, autonomy and interaction. Moreover, the data are standard by maintaining some important criteria. The methodology for collecting data is shown in the Table 2. Every data type has similar content and format, so they have internal consistency, and concurrent validity. Table 3 reveals the details of above mentioned four types of datasets.

**Table 2:** Methodology of data collection.

Research design	Location	Sample	Data collection procedures	Data analysis procedures
Mixed methods (explanatory)	Peninsular Malaysia	954	Focus group discussion and questionnaires	Qualitative (descriptive analysis), Statistical Package for Social Sciences (SPSS) and Receiver Operating Characteristic (ROC)

**Table 3:** Four types of autism datasets including instances, cut-off score, sensitivity, specificity, and validity.

ASQ:SE (M)	Instances (n)	Cut-off score	Sensitivity	Specificity	Agreement (%)
24 months	209	71	83	98	90.0
30 months	209	95	71	98	95.0
36 months	234	100	67	94	96.0
48 months	302	105	73	95	98.0

*ASQ:SE (M)* Ages & Stages Questionnaire: Social Emotional (Malaysia).

### Simulation setup

The implementation of the recommended methodology was carried out utilizing Python programming platform. The above datasets were employed for this experiments. However, the performances of the proposed framework were measured by some objective functions as parameters, they were the information hiding failure rate (*C*_1_), the information preservation rate (*C*_2_), the degree of modification rate (*C*_3_), and the fitness function (*f*). Furthermore, the performances were measured by those parameters by setting the different values of the acceleration constants (*c*_1,_ *c*_2_), where 0 < (*c*_1,_ *c*_2_) <  = 1.

### Results and discussions

"Results and discussions"(a) illustrated the achieved performances of the proposed Enhanced Combined PSO-GWO restoration process versus the other traditional algorithms against attacks, namely Particle Swarm Optimization (PSO), Genetic Algorithm (GA), Differential Evolution (DE), Crow Search Algorithm (CSA), and Adaptive Awareness Probability-based CSA (AAP-CSA). Following that, the effectiveness of this framework was demonstrated by varying the acceleration constants *c*_1_ and *c*_2_ in "Results and discussions"(b).

#### Comparison with traditional algorithms

Tables 4, 5, 6, and 7 demonstrate the performance analysis by restoration procedure of the Enhanced Combined PSO-GWO framework for the four autism datasets.

**Table 4:** Analysis of data restoration performance for dataset of 24 months autism child.

Functions	GA^40^	PSO^41^	CSA^42^	AAP-CSA^43^	DE^44^	PSO-GWO
*C*_1_	7.333332	6.73110417	2.255556	0.723232	8.666669	0.049582688
*C*_2_	0.985866	0.96665588	0.899989	1.002099	0.899999	1.007415858
*C*_3_	2199.030	450.040250	400.0021	490.3019	470.5105	610.5032997
*f*	3.906688	31.1909070	0.713161	60.10929	19.50060	33.89987687

**Table 5:** Analysis of data restoration performance for dataset of 30 months autism child.

Functions	GA^40^	PSO^41^	CSA^42^	AAP-CSA^43^	DE^44^	PSO-GWO
*C*_1_	2.57553	4.45	0.791470	2.11810	1.096949	0.005785754
*C*_2_	0.999245	0.8922342	2.005758	0.899899	0.898999	1.031786858
*C*_3_	2390.050	1210.2510	1560.998	1055.604	1310.297	1410.203456
*f*	22.43441	31.2160208	4.521818	49.60847	59.50199	65.88978746

**Table 6:** Analysis of data restoration performance for 36 months autism child dataset.

Functions	GA^40^	PSO^41^	CSA^42^	AAP-CSA^43^	DE^44^	PSO-GWO
*C*_1_	0.089899	0.80	0.089989	2.252020	0.6	0.079456492
*C*_2_	2.100122	2.00092711	2.115234	0.889812	2.112323	1.020892055
*C*_3_	9040.232	6299.98897	5999.876	4499.201	8001.009	8250.055682
*f*	17.42103	150.080677	40.06892	180.3530	400.7061	105.4525589

**Table 7:** Analysis of data restoration performance for 48 months autism child dataset.

Functions	GA^40^	PSO^41^	CSA^42^	AAP-CSA^43^	DE^44^	PSO-GWO
*C*_1_	0.6	0.0919090	0.3	0.4	0.2	0.089876584
*C*_2_	2.090031	2.0033536	2.022078	2.009948	2.009987	1.095866585
*C*_3_	4010.120	13,120.440	8570.42	9714.452	9289.420	8980.896565
*f*	20.39320	150.896020	10.799127	400.1057	350.6099	115.9087576

Initially, for a 24-month autism child dataset, the proposed algorithm performs 99.26%, 99.43%, 99.32%, 93.14%, and 97.80% better than PSO, DE, GA, AAP-CSA, and CSA for *C*_1_ as shown in Table 4. This model also outperforms GA for *C*_3_ by 72.24%. Furthermore, the recommended approach outperforms AAP-CSA by 43.60% for *f*.

Table 5 compares the performance of the proposed framework for 30 months autism child dataset to the existing algorithms. The framework reveals 99.78%, 99.87%, 99.27%, 99.47%, and 99.73% enhancement over GA, PSO, CSA, DE, and AAP-CSA algorithms respectively, for *C*_1_. The framework also attains 48.56% greater than CSA for *C*_2_. Furthermore, regarding *C*_3_, the proposed model outperforms GA and CSA by 41% and 9.66%.

Table 6 illustrates the performance improvements of the proposed model when applied to a 36-month autism child dataset in terms of *C*_1_, which is 11.62%, 90.07%, 11.70%, 86.76%, and 96.47% superior to GA, PSO, CSA, DE, and AAP-CSA algorithms individually. Again, this model outperforms PSO, GA, DE, and CSA by 48.98%, 51.39%, 51.67%, 51.74%, respectively for *C*_2_, whereas GA by 8.74% for *C*_3_. Following that, the model also outperforms PSO, DE, and AAP-CSA algorithms by 29.74%, 73.68%, and 41.53% respectively, in the case of *f*.

Finally, Table 7 shows that, the proposed model outperforms GA, PSO, CSA, DE, and AAP-CSA algorithms by 85.02%, 2.21%, 70.04%, 55.06%, and 77.53%, respectively, in terms of *C*_1_ for the 48-month autism child dataset. The model attains 47.57%, 45.30%, 45.80%, 45.48%, 45.48% over GA, PSO, CSA, DE, AAP-CSA, respectively for *C*_2_ In terms of *C*_3_, the model outperforms the DE, PSO, AAP-CSA algorithms by 3.32%, 31.55%, 7.55%, respectively. In the case of *f*, the proposed framework outperforms PSO, DE, and AAP-CSA models by 23.19%, 66.94%, and 71.03%, respectively.

In summary, the restoration process of the proposed model outperforms other traditional algorithms, as evidenced by the tabular results presented above.

#### Effectiveness of enhanced combined PSO-GWO based on acceleration constants c_1_ and c_2_

**Figure 4:**
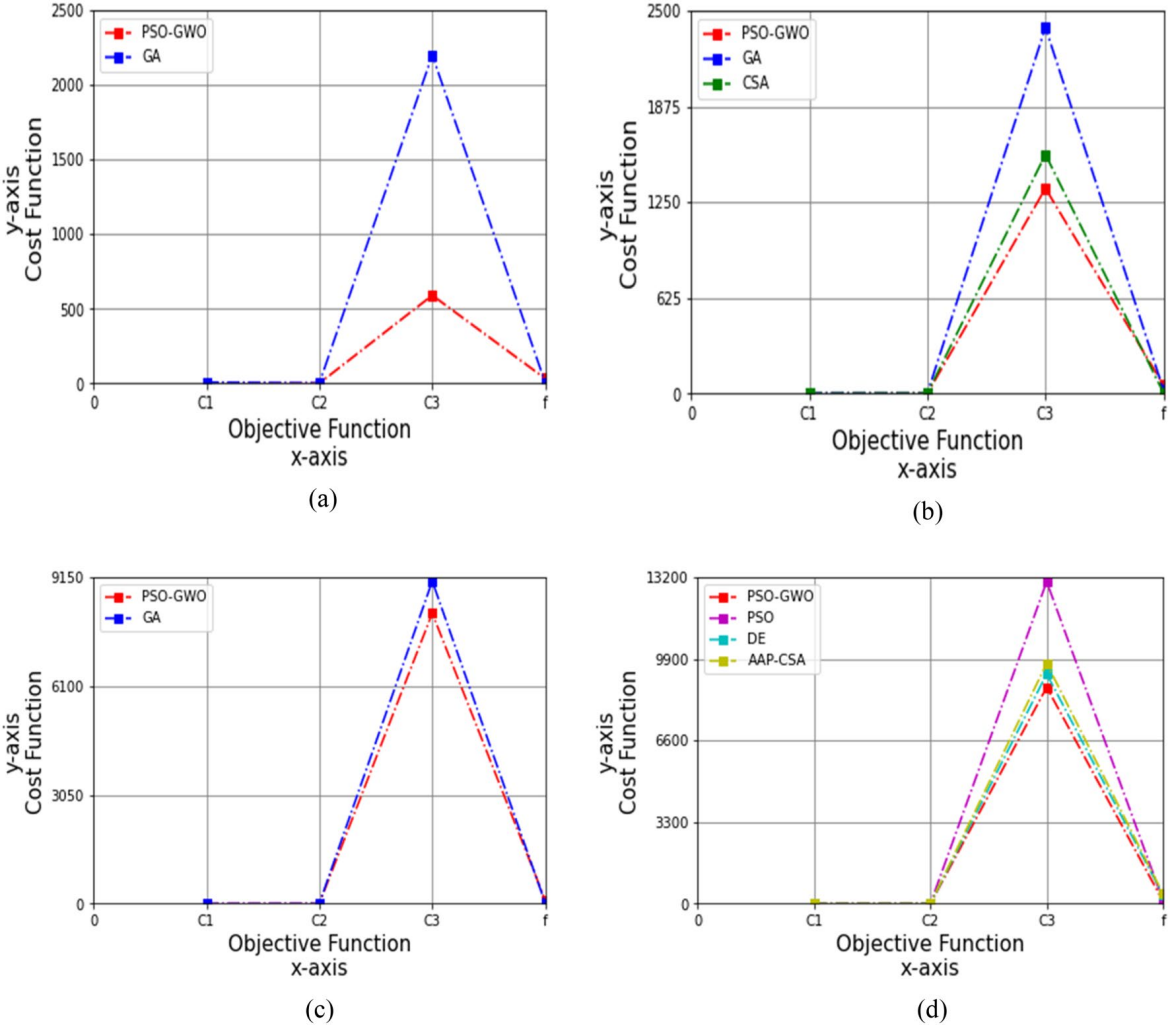
Performance analysis on cost function for four datasets, (**a**) autism child dataset 24 months, (**b**) autism child dataset 30 months, (**c**) autism child dataset 36 months, (**d**) autism child dataset 48 months, while *c*_1_ = 0.1 and *c*_2_ = 0.1.

**Figure 5:**
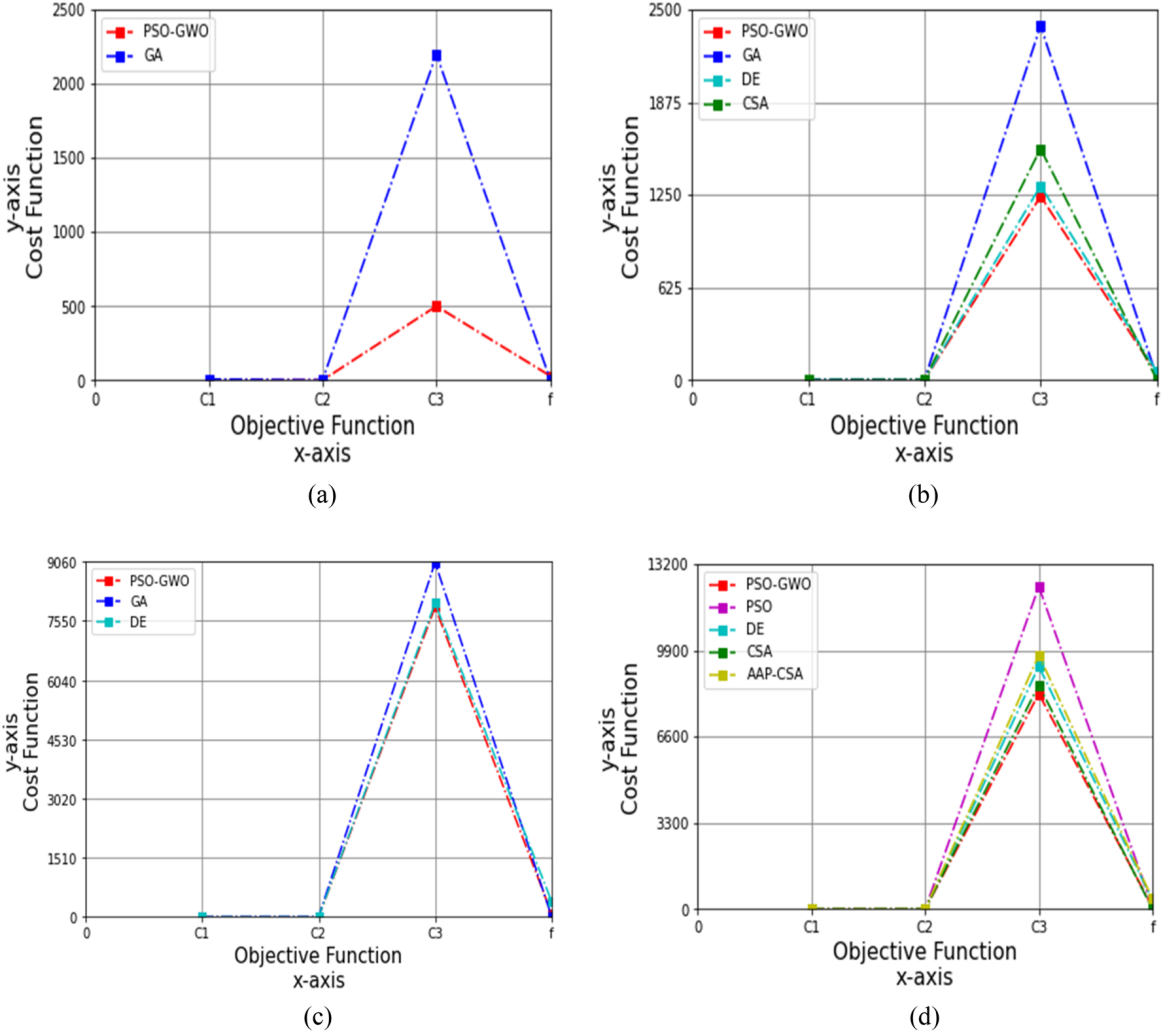
Performance analysis on cost function for four datasets, (**a**) autism child dataset 24 months, (**b**) autism child dataset 30 months, (**c**) autism child dataset 36 months, (**d**) autism child dataset 48 months, while *c*_1_ = 0.3 and *c*_2_ = 0.3.

**Figure 6:**
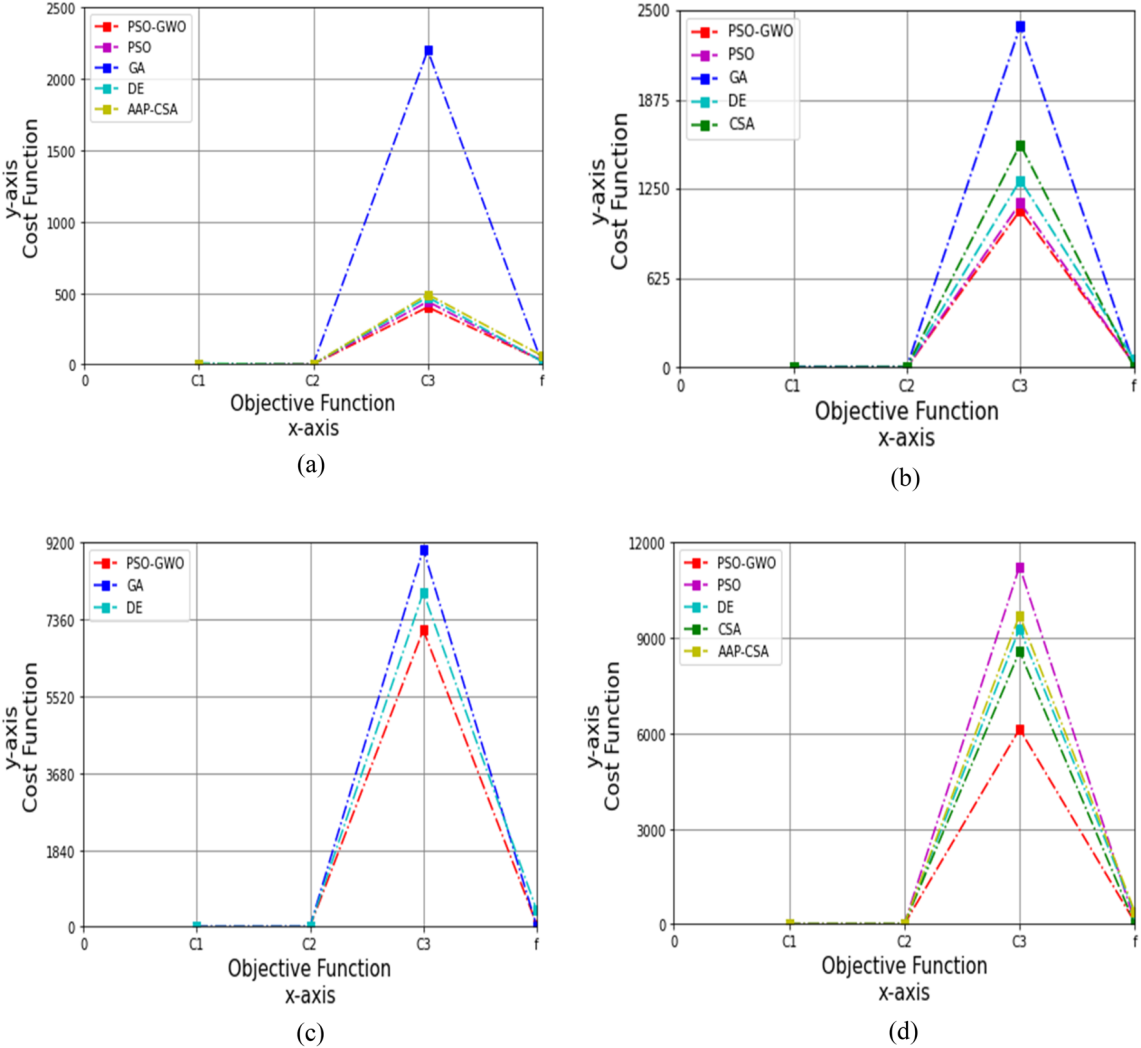
Performance analysis on cost function for four datasets, (**a**) autism child dataset 24 months, (**b**) autism child dataset 30 months, (**c**) autism child dataset 36 months, (**d**) autism child dataset 48 months, while *c*_1_ = 0.5 and *c*_2_ = 0.5.

**Figure 7:**
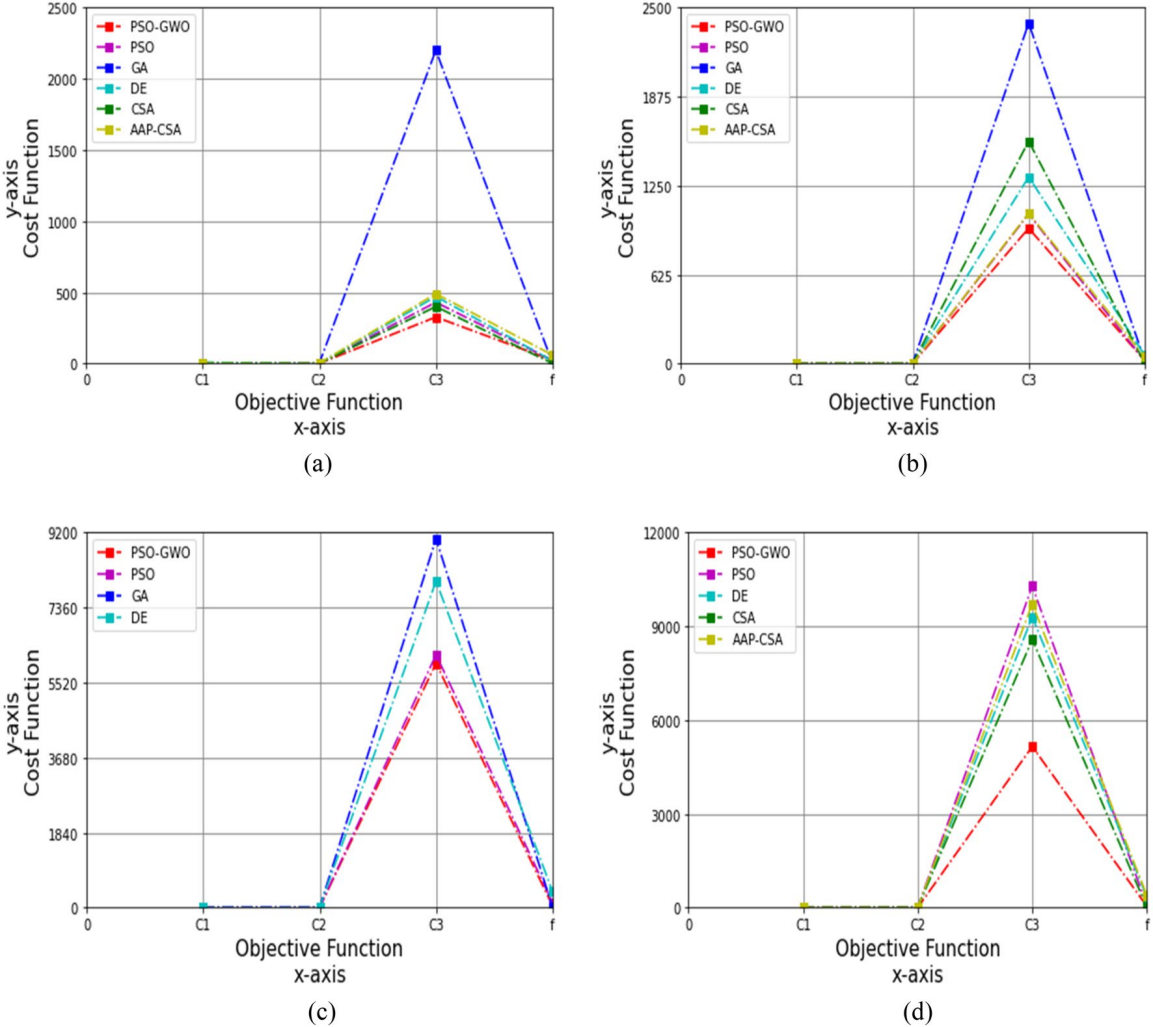
Performance analysis on cost function for four datasets, (**a**) autism child dataset 24 months, (**b**) autism child dataset 30 months, (**c**) autism child dataset 36 months, (**d**) autism child dataset 48 months, while *c*_1_ = 0.7 and *c*_2_ = 0.7.

**Figure 8:**
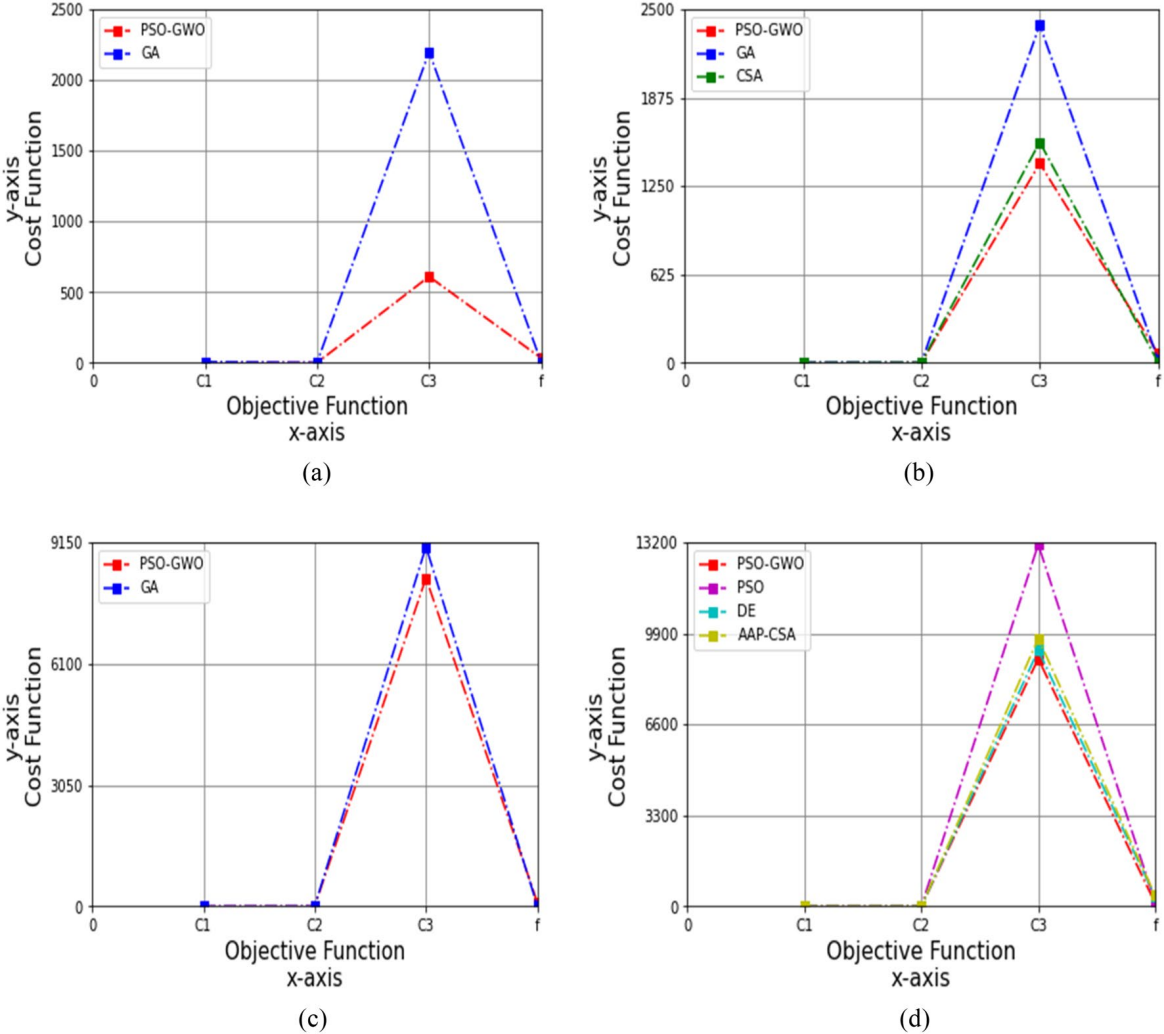
Performance analysis on cost function for four datasets, (**a**) autism child dataset 24 months, (**b**) autism child dataset 30 months, (**c**) autism child dataset 36 months, (**d**) autism child dataset 48 months, while *c*_1_ = 1 and *c*_2_ = 1.

The restoration of autism data has been measured based on objective functions. Here, the acceleration constants have been updated by varying the values their values between 0 and 1 to satisfy the condition, 0 < (*c*_1,_ *c*_2_) <  = 1. Figures 4, 5, 6, 7 and 8 graphically illustrate the results of performance analysis on cost functions obtained by Eq. (8) for four types of autism child datasets based on these varying values.

i) The acceleration constants, *c*_1_ = 0.1 and *c*_2_ = 0.1

At first, by taking the 24 months autism child dataset with the values for *c*_1_ = 0.1 and *c*_2_ = 0.1, the outcomes of GA for objective functions *C*_1_, *C*_2_, *C*_3_, and *f* are 7.333, 0.985, 2199.030, and 3.906, while the proposed technique achieves 0.049, 1.007, 590.40, and 32.80, correspondingly, as shown in Table 8. And the simulation is shown in Fig. 4a, where the proposed technique is 99.33%, 73.15% more improved than GA for *C*_1_, and *C*_3_.

**Table 8:** Cost analysis for 24 months autism data, while *c*_1_ = 0.1 and *c*_2_ = 0.1

Functions	GA	PSO-GWO
*C*_1_	7.333	0.049
*C*_2_	0.985	1.007
*C*_3_	2199.030	590.40
*f*	3.906	32.80

Similarly, the values of *f, C*_1_, *C*_3_, and *C*_2_ for GA are 22.434, 2.575, 2390.050, and 0.999, whereas CSA is 4.521, 0.791, 1560.998, and 2.005, and the proposed method is 55.20, 0.005, 1340.10, and 1.031, respectively, for the 30 months autism child dataset, summarized in Table 9. So, the proposed technique shows 99.81% and 43.93% higher than GA for *C*_1_, and *C*_3_ correspondingly, whereas 99.37%, 48.58%, and 14.15% greater than CSA for *C*_1_, *C*_2_, and *C*_3_, respectively, which are simulated in Fig. 4b.

**Table 9:** Cost analysis for 30 months autism data, while *c*_1_ = 0.1 and *c*_2_ = 0.1

Functions	GA	CSA	PSO-GWO
*C*_1_	2.575	0.791	0.005
*C*_2_	0.999	2.005	1.031
*C*_3_	2390.050	1560.998	1340.10
*f*	22.434	4.521	55.20

For the 36 months autism child dataset, the objective functions *C*_1_, *C*_2_, *C*_3_, and *f* for the GA are 0.089, 2.100, 9040.232, and 17.421, whereas the proposed scheme yielded 0.079, 1.020, 8140.45, and 100.30, correspondingly, as revealed in Table 10. Figure 4c illustrates that the proposed method is 11.24%, 51.43%, and 9.95% enhanced over GA for *C*_1_, *C*_2_, and *C*_3_.

**Table 10:** Cost analysis for 36 months autism data, while *c*_1_ = 0.1 and *c*_2_ = 0.1

Functions	GA	PSO-GWO
*C*_1_	0.089	0.079
*C*_2_	2.100	1.020
*C*_3_	9040.232	8140.45
*f*	17.421	100.30

In case of 48 months autism child dataset, the *C*_1_, *C*_2_, *C*_3_, and *f* of PSO are 0.091, 2.003, 13,020.30, and 140.60, respectively; DE are 0.2, 2.009, 9289.420, and 350.609, and AAP-CSA are 0.4, 2.009, 9714.452, and 400.105; whereas the proposed framework attains 0.089, 1.095, 8750.40, and 105.80, respectively, as shown in Table 11.

**Table 11:** Cost analysis for 48 months autism data, while *c*_1_ = 0.1 and *c*_2_ = 0.1

Functions	PSO	DE	AAP-CSA	PSO-GWO
*C*_1_	0.091	0.2	0.4	0.089
*C*_2_	2.003	2.009	2.009	1.095
*C*_3_	13,020.30	9289.420	9714.452	8750.40
*f*	140.60	350.609	400.105	105.80

Figure 4d shows that the PSO-GWO is higher than PSO by 2.20%, 45.33%, 32.79%, and 24.75%; DE by 55.5%, 45.50%, 5.80%, and 69.82%; and AAP-CSA by 77.75%, 45.50%, 9.92%, and 73.56% for *C*_1_, *C*_2_, *C*_3_, and *f*, respectively.

ii) The acceleration constants, *c*_1_ = 0.3 and *c*_2_ = 0.3

After that, the values of the acceleration constants are set to *c*_1_ = 0.3 and *c*_2_ = 0.3, so the objective functions, *C*_1_, *C*_2_, *C*_3_, and *f* for GA are 7.333, 0.985, 2199.030, and 3.906, while the proposed technique achieves 0.049, 1.007, 500.25, and 30.05, respectively, over the 24 months autism child dataset, shown in Table 12. The simulation results are graphically presented in Fig. 5a, where the proposed technique is 99.33%, or 77.25%, more improved than GA for *C*_1_ and *C*_3_.

**Table 12:** Cost analysis for 24 months autism data, while *c*_1_ = 0.3 and *c*_2_ = 0.3

Functions	GA	PSO-GWO
*C*_1_	7.333	0.049
*C*_2_	0.985	1.007
*C*_3_	2199.030	500.25
*f*	3.906	30.05

Under the 30 months autism child dataset, the values of *C*_1_, *C*_2_, *C*_3_, and *f* for GA are 2.575, 0.999, 2390.050, and 22.434, DE are 1.096, 0.898, 1310.297, and 59.501, and CSA are 0.791, 2.005, 1560.998, and 4.521, whereas the proposed method attains 0.005, 1.031, 1240.10, and 45.15, respectively, as depicted in Table 13. The simulation results are revealed in Fig. 5b. Here, the suggested method is 99.81% and 48.11% superior to GA for *C*_1_ and *C*_3_, and 99.54%, 5.36%, and 24.12% better than DE for *C*_1_, *C*_3_, and *f*, as well as 99.37%, 48.58%, and 20.56% greater than CSA for *C*_1_, *C*_2_, and *C*_3_.

**Table 13:** Cost analysis for 30 months autism data, while *c*_1_ = 0.3 and *c*_2_ = 0.3

Functions	GA	DE	CSA	PSO-GWO
*C*_1_	2.575	1.096	0.791	0.005
*C*_2_	0.999	0.898	2.005	1.031
*C*_3_	2390.050	1310.297	1560.998	1240.10
*f*	22.434	59.501	4.521	45.15

For the 36 months autism child dataset, the objective functions *C*_1_, *C*_2_, *C*_3_, and *f* for the GA are 0.089, 2.100, 9040.232, and 17.421, and the DE are 0.6, 2.112, 8001.009, and 400.706, wherein the attaining outcomes are 0.079, 1.020, 7900.25, and 90.20 correspondingly, as presented in Table 14. The proposed method is 11.24%, 51.43%, and 12.61% higher than GA for *C*_1_, *C*_2_, and *C*_3_. Furthermore, the method yielded 86.83%, 51.70%, 1.26%, and 77.49% greater than DE for *C*_1_, *C*_2_, *C*_3_, and *f*, as demonstrated in Fig. 5c.

**Table 14:** Cost analysis for 36 months autism data, while *c*_1_ = 0.3 and *c*_2_ = 0.3

Functions	GA	DE	PSO-GWO
*C*_1_	0.089	0.6	0.079
*C*_2_	2.100	2.112	1.020
*C*_3_	9040.232	8001.009	7900.25
*f*	17.421	400.706	90.20

Under the 48 months autism child dataset, the results of *C*_1_, *C*_2_, *C*_3_, and *f* for PSO are 0.091, 2.003, 12,340.20, and 100.40, respectively; DE are 0.2, 2.009, 9289.420, and 350.609; CSA are 0.3, 2.022, 8570.42, and 10.799; and AAP-CSA are 0.4, 2.009, 9714.452, and 400.105; whereas the suggested framework achieves 0.089, 1.095, 8240.20, and 85.60, respectively, represented in Table 15.

**Table 15:** Cost analysis for 48 months autism data, while *c*_1_ = 0.3 and *c*_2_ = 0.3

Functions	PSO	DE	CSA	AAP-CSA	PSO-GWO
*C*_1_	0.091	0.2	0.3	0.4	0.089
*C*_2_	2.003	2.009	2.022	2.009	1.095
*C*_3_	12,340.20	9289.420	8570.42	9714.452	8240.20
*f*	100.40	350.609	10.799	400.105	85.60

The simulation results are graphically illustrated in Fig. 5d and revealed that this technique yielded 2.20%, 45.33%, 33.22%, and 14.74% higher than PSO, 55.5%, 45.50%, 11.29%, and 75.59% greater than DE, 77.75%, 45.50%, 15.18%, and 78.61% superior to AAP-CSA for *C*_1_, *C*_2_, *C*_3_, and *f*, whereas 70.33%, 45.85%, and 3.85% were better than CSA for *C*_1_, *C*_2_, and *C*_3_, respectively.

iii) The acceleration constants, *c*_1_ = 0.5 and *c*_2_ = 0.5

Correspondingly, for the 24 months autism child dataset in case of *c*_1_ = 0.5 and *c*_2_ = 0.5, the outcomes of *C*_1_, *C*_2_, *C*_3_, and *f* for PSO are 6.731, 0.966, 440.10, and 24.15, GA are 7.333, 0.985, 2199.030, and 3.906, DE are 8.666, 0.899, 470.51, and 19.5, and AAP-CSA are 0.723, 1.002, 490.301, and 60.109, while the proposed technique achieves 0.049, 1.007, 401.15, and 25.10, respectively, as shown in Table 16. Figure 6a demonstrates that the suggested method is 99.27%, 8.85% better than PSO, 99.33%, 81.76% higher than GA, 99.43%, 14.74% superior to DE for *C*_1_, and *C*_3_, whereas 93.22%, 18.18%, and 58.24% greater than AAP-CSA for *C*_1_, *C*_3_, and *f*, respectively.

**Table 16:** Cost analysis for 24 months autism data, while *c*_1_ = 0.5 and *c*_2_ = 0.5

Functions	PSO	GA	DE	AAP-CSA	PSO-GWO
*C*_1_	6.731	7.333	8.666	0.723	0.049
*C*_2_	0.966	0.985	0.899	1.002	1.007
*C*_3_	440.10	2199.030	470.51	490.301	401.15
*f*	24.15	3.906	19.5	60.109	25.10

Moreover, for the 30 months autism child dataset, the values of *C*_1_, *C*_2_, *C*_3_, and *f* for PSO are 4.45, 0.892, 1150.15, and 25.11, GA are 2.575, 0.999, 2390.050, and 22.434, DE are 1.096, 0.898, 1310.297, and 59.501, and CSA are 0.791, 2.005, 1560.998, and 4.521, whereas the proposed method conquers 0.005, 1.031, 1100.20, and 30.25, respectively, represented in Table 17. The simulation is revealed in Fig. 6b. Here, the recommended technique is 99.89%, 4.34% greater than PSO, 99.81%, and 53.97% superior to GA for *C*_1_ and *C*_3_, and 99.54%, 16.03%, and 49.16% better than DE for *C*_1_, *C*_3_, and *f*, as well as 99.37%, 48.58%, and 29.52% greater than CSA for *C*_1_, *C*_2_, and *C*_3_, respectively.

**Table 17:** Cost analysis for 30 months autism data, while *c*_1_ = 0.5 and *c*_2_ = 0.5

Functions	PSO	GA	DE	CSA	PSO-GWO
*C*_1_	4.45	2.575	1.096	0.791	0.005
*C*_2_	0.892	0.999	0.898	2.005	1.031
*C*_3_	1150.15	2390.050	1310.297	1560.998	1100.20
*f*	25.11	22.434	59.501	4.521	30.25

Applying the 36 months autism child dataset, the objective functions *C*_1_, *C*_2_, *C*_3_, and *f* for the GA are 0.089, 2.100, 9040.232, and 17.421, and the DE are 0.6, 2.112, 8001.009, and 400.706, wherein the attaining outcomes are 0.079, 1.020, 7100.45, and 65.10, correspondingly, as presented in Table 18. The suggested scheme is 11.24%, 51.43%, and 21.46% higher than GA for *C*_1_, *C*_2_, and *C*_3_, wherein 86.83%, 51.70%, 11.26%, and 83.75% greater than DE for *C*_1_, *C*_2_, *C*_3_, and *f*, respectively, which are demonstrated in Fig. 6c.

**Table 18:** Cost analysis for 36 months autism data, while *c*_1_ = 0.5 and *c*_2_ = 0.5

Functions	GA	DE	PSO-GWO
*C*_1_	0.089	0.6	0.079
*C*_2_	2.100	2.112	1.020
*C*_3_	9040.232	8001.009	7100.45
*f*	17.421	400.706	65.10

Furthermore, the results of *C*_1_, *C*_2_, *C*_3_, and *f* for PSO are 0.091, 2.003, 11,245.10, and 85.30, respectively; DE are 0.2, 2.009, 9289.420, and 350.609; CSA are 0.3, 2.022, 8570.42, and 10.799; and AAP-CSA are 0.4, 2.009, 9714.452, and 400.105; whereas the suggested framework achieves 0.089, 1.095, 6130.10, and 65.30, respectively, applying the 48 months autism child dataset, shown in Table 19.

**Table 19:** Cost analysis for 48 months autism data, while *c*_1_ = 0.5 and *c*_2_ = 0.5

Functions	PSO	DE	CSA	AAP-CSA	PSO-GWO
*C*_1_	0.091	0.2	0.3	0.4	0.089
*C*_2_	2.003	2.009	2.022	2.009	1.095
*C*_3_	11,245.10	9289.420	8570.42	9714.452	6130.10
*f*	85.30	350.609	10.799	400.105	65.30

The simulation is demonstrated in Fig. 6d, and it is revealed that this technique is 2.20%, 45.33%, 45.49%, and 23.45% higher than PSO, 55.5%, 45.50%, 34%, and 81.38% greater than DE, 77.75%, 45.50%, 36.90%, and 83.68% superior to AAP-CSA for *C*_1_, *C*_2_, *C*_3_, and *f*, whereas 70.33%, 45.85%, and 28.47% better than CSA for *C*_1_, *C*_2_, and *C*_3_, respectively.

iv) The acceleration constants, *c*_1_ = 0.7 and *c*_2_ = 0.7

Similarly, considering the 24 months autism child dataset to set *c*_1_ = 0.7 and *c*_2_ = 0.7, the outcomes of *C*_1_, *C*_2_, *C*_3_, and *f* for PSO are 6.731, 0.966, 430.10, and 23.10; GA are 7.333, 0.985, 2199.030, and 3.906; DE are 8.666, 0.899, 470.51, and 19.5; CSA are 2.255, 0.899, 400.002, and 0.71; and AAP-CSA are 0.723, 1.002, 490.301, and 60.109, while the proposed technique achieves 0.049, 1.007, 326.25, and 24.05, respectively, summarized in Table 20. Figure 7a demonstrates that the suggested method is 99.27%, 24.15% better than PSO, 99.33%, 85.16% higher than GA, 99.43%, 30.66% superior to DE, 97.83%, 18.44% more enhanced over CSA for *C*_1_, and *C*_3_, whereas 93.22%, 33.46%, and 59.99% greater than AAP-CSA for *C*_1_, *C*_3_, and *f*, respectively.

**Table 20:** Cost analysis for 24 months autism data, while *c*_1_ = 0.7 and *c*_2_ = 0.7

Functions	PSO	GA	DE	CSA	AAP-CSA	PSO-GWO
*C*_1_	6.731	7.333	8.666	2.255	0.723	0.049
*C*_2_	0.966	0.985	0.899	0.899	1.002	1.007
*C*_3_	430.10	2199.030	470.51	400.002	490.301	326.25
*f*	23.10	3.906	19.5	0.713	60.109	24.05

Using a 30 months autism child dataset, the results of *C*_1_, *C*_2_, *C*_3_, and *f* for PSO are 4.45, 0.892, 1050.35, and 20.15; GA are 2.575, 0.999, 2390.050, and 22.434; DE are 1.096, 0.898, 1310.297, and 59.501; CSA are 0.791, 2.005, 1560.998, and 4.521; and AAP-CSA are 2.118, 0.899, 1055.604, and 49.608, whereas the proposed method conquers 0.005, 1.031, 950.30, and 23.15, respectively, as revealed in Table 21. The simulation is illustrated in Fig. 7b. So, the recommended technique is 99.89% and 9.53% greater than PSO, 99.81%, and 60.24% superior to GA for *C*_1_ and *C*_3_, and 99.54%, 27.47%, and 61.09% better than DE, 99.76%, 9.98%, and 53.33% more improved over AAP-CSA for *C*_1_, *C*_3_, and *f*, as well as 99.37%, 48.58%, and 39.12% greater than CSA for *C*_1_, *C*_2_, and *C*_3_, respectively.

**Table 21:** Cost analysis for 30 months autism data, while *c*_1_ = 0.7 and *c*_2_ = 0.7

Functions	PSO	GA	DE	CSA	AAP-CSA	PSO-GWO
*C*_1_	4.45	2.575	1.096	0.791	2.118	0.005
*C*_2_	0.892	0.999	0.898	2.005	0.899	1.031
*C*_3_	1050.35	2390.050	1310.297	1560.998	1055.604	950.30
*f*	20.15	22.434	59.501	4.521	49.608	23.15

For the 36 months autism child dataset, the results of *C*_1_, *C*_2_, *C*_3_, and *f* for PSO are 0.80, 2.00, 6199.15, and 130.10, GA are 0.089, 2.100, 9040.232, and 17.421; DE are 0.6, 2.112, 8001.009, and 400.706; wherein the attaining outcomes are 0.079, 1.020, 6115.15, and 45.25, correspondingly, as represented in Table 22. The suggested scheme is 90.13%, 49%, 3.37%, and 65.22% superior to PSO for *C*_1_, *C*_2_, *C*_3_, and *f*, 11.24%, 51.43%, and 33.74% higher than GA for *C*_1_, *C*_2_, and *C*_3_, wherein 86.83%, 51.70%, 25.13%, and 88.71% greater than DE for *C*_1_, *C*_2_, *C*_3_, and *f*, respectively, which are demonstrated in Fig. 7c.

**Table 22:** Cost analysis for 36 months autism data, while *c*_1_ = 0.7 and *c*_2_ = 0.7

Functions	PSO	GA	DE	PSO-GWO
*C*_1_	0.80	0.089	0.6	0.079
*C*_2_	2.00	2.100	2.112	1.020
*C*_3_	6199.15	9040.232	8001.009	5990.15
*f*	130.10	17.421	400.706	45.25

Under the 48 months autism dataset, the objective functions *C*_1_, *C*_2_, *C*_3_, and *f* for PSO are 0.091, 2.003, 10,280.20, and 65.20, respectively; DE are 0.2, 2.009, 9289.420, and 350.609; CSA are 0.3, 2.022, 8570.42, and 10.799; and AAP-CSA are 0.4, 2.009, 9714.452, and 400.105; whereas the suggested framework achieves 0.089, 1.095, 5150.20, and 45.20, respectively, shown in Table 23.

**Table 23:** Cost analysis for 48 months autism data, while *c*_1_ = 0.7 and *c*_2_ = 0.7

Functions	PSO	DE	CSA	AAP-CSA	PSO-GWO
*C*_1_	0.091	0.2	0.3	0.4	0.089
*C*_2_	2.003	2.009	2.022	2.009	1.095
*C*_3_	10,280.20	9289.420	8570.42	9714.452	5150.20
*f*	65.20	350.609	10.799	400.105	45.20

The simulation is demonstrated in Fig. 7d, and it is revealed that this technique is 2.20%, 45.33%, 49.90%, and 30.67% higher than PSO, 55.5%, 45.50%, 44.56%, and 87.11% greater than DE, 77.75%, 45.50%, 46.98%, and 88.70% superior to AAP-CSA for *C*_1_, *C*_2_, *C*_3_, and *f*, whereas 70.33%, 45.85%, and 39.91% are better than CSA for *C*_1_, *C*_2_, and *C*_3_, respectively.

v) The acceleration constants, *c*_1_ = 1 and *c*_2_ = 1.

Moreover, when considering the values of acceleration constants as *c*_1_ = 1 and *c*_2_ = 1 and using a dataset of autism children spanning over a period of 24 months, the GA yields results of 7.333, 0.985, 2199.030, and 3.906 for *C*_1_, *C*_2_, *C*_3_, and *f*, respectively. However, the proposed framework produces outcomes of 0.049, 1.007, 610.503, and 33.899 for the same parameters, as depicted in Table 24. Notably, the recommended framework shows a 99.33% and 72.24% improvement over GA for *C*_1_ and *C*_3_, respectively. The simulation results are illustrated in Fig. 8a.

**Table 24:** Cost analysis for 24 months autism data, while *c*_1_ = 1 and *c*_2_ = 1.

Functions	GA	PSO-GWO
*C*_1_	7.333	0.049
*C*_2_	0.985	1.007
*C*_3_	2199.030	610.503
*f*	3.906	33.899

For the 30 months autism child dataset, the values of objective functions *C*_1_, *C*_2_, *C*_3_, and *f* for GA are 2.575, 0.999, 2390.050, and 22.434, whereas CSA is 0.791, 2.005, 1560.998, and 4.521, and the proposed method is 0.005, 1.031, 1410.203, and 65.889, respectively, summarized in Table 25. The simulation is shown in Fig. 8b. The proposed technique is 99.81%, 41% over GA for *C*_1_ and *C*_3_, correspondingly, and 99.37%, 48.58%, and 9.66% enhanced over CSA for *C*_1_, *C*_2_, and *C*_3_, respectively.

**Table 25:** Cost analysis for 30 months autism data, while *c*_1_ = 1 and *c*_2_ = 1.

Functions	GA	CSA	PSO-GWO
*C*_1_	2.575	0.791	0.005
*C*_2_	0.999	2.005	1.031
*C*_3_	2390.050	1560.998	1410.203
*f*	22.434	4.521	65.889

Similarly, the GA are 0.089, 2.100, 9040.232, and 17.421, wherein the proposed scheme assessed 0.079, 1.020, 8250.055, and 105.452 for *C*_1_, *C*_2_, *C*_3_, and *f*, respectively, under the 36 months autism dataset, as presented in Table 26. The simulation is shown in Fig. 8c. Here, the proposed scheme is 11.24%, 51.43%, and 8.74% greater than GA for *C*_1_, *C*_2_, and *C*_3_.

**Table 26:** Cost analysis for 36 months autism data, while *c*_1_ = 1 and *c*_2_ = 1.

Functions	GA	PSO-GWO
*C*_1_	0.089	0.079
*C*_2_	2.100	1.020
*C*_3_	9040.232	8250.055
*f*	17.421	105.452

Finally, for the 48 months autism child dataset, the values of objective functions *C*_1_, *C*_2_, *C*_3_, and *f* of PSO are 0.091, 2.003, 13,120.440, and 150.896, respectively; DE are 0.2, 2.009, 9289.420, and 350.609, whereas AAP-CSA are 0.4, 2.009, 9714.452, and 400.105; and the suggested framework achieved 0.089, 1.095, 8980.896, and 115.908, shown in Table 27.

**Table 27:** Cost analysis for 48 months autism data, while *c*_1_ = 1 and *c*_2_ = 1.

Functions	PSO	DE	AAP-CSA	PSO-GWO
*C*_1_	0.091	0.2	0.4	0.089
*C*_2_	2.003	2.009	2.009	1.095
*C*_3_	13,120.440	9289.420	9714.452	8980.896
*f*	150.896	350.609	400.105	115.908

The simulation is shown in Fig. 8d, where PSO-GWO is higher than PSO by 2.20%, 45.33%, 31.55%, and 23.19%; DE by 55.5%, 45.50%, 3.32%, and 66.94%; and AAP-CSA by 77.75%, 45.50%, 7.55%, and 71.03% for *C*_1_, *C*_2_, *C*_3_, and *f*.

## Discussions

The proposed restoration process was used to restore the data, and its performance was compared to the existing techniques. The results, which are discussed in "Results and discussions"(a), show that the proposed technique outperforms the existing techniques. Subsequently, the performance of the proposed technique was further improved by varying the acceleration constants within the range of 0 < (*c*_1_, *c*_2_) <  = 1, as shown in "Results and discussions"(b).

For instance, for 24 months of autism data, the technique performs 99.26%, 99.32%, 99.43%, 97.80%, and 93.14% greater than PSO, GA, DE, CSA, and AAP-CSA for *C*_1_, 72.24% better than GA for *C*3, and 43.60% better than AAP-CSA for *f*, whereas by the values *c*_1_ = 0.7 and c_2_ = 0.7, the technique achieves 99.27%, 99.33%, 99.43%, 97.83%, and 93.22% higher than PSO, GA, DE, CSA, and AAP-CSA for *C*_1_, 24.15%, 85.16%, 30.66%, 18.44%, and 33.46% greater than PSO, GA, DE, CSA, and AAP-CSA for *C*3, and 59.99% better than AAP-CSA for *f*. Additionally, for 30 months of autism data, the technique performs 99.87%, 99.78%, 99.47%, 99.27%, and 99.73% enhanced over PSO, GA, DE, CSA, and AAP-CSA, correspondingly, for *C*_1_, 41% and 9.66% improved over GA and CSA, respectively, for *C*3, wherein at *c*_1_ = 0.7 and *c*_2_ = 0.7, the technique achieves 99.89%, 99.81%, 99.54%, 99.37%, and 99.76% higher than PSO, GA, DE, CSA, and AAP-CSA for *C*_1_, 9.53%, 60.24%, 27.47%, 39.12%, and 9.98% greater than PSO, GA, DE, CSA, and AAP-CSA for *C*3, and also 61.09%, 53.33% greater than DE and AAP-CSA, respectively for *f*. Furthermore, the results indicate that when the values of acceleration constants are set to 0.7, the proposed technique performs comparably with the existing methods for the 36- and 48-month autism datasets. Similarly, when various values for *c*_1_ and *c*_2_, 0 < (*c*_1_, *c*_2_) <  = 1, are used with different types of autism data, the proposed technique demonstrates superior performance.

Therefore, this research work has some significant implications for different arena. Nowadays, healthcare organisations are becoming increasingly concerned about the possibility of personal health data breaches as a result of the quick proliferation of digital health technology. Healthcare data is extremely vulnerable because of cyber hackers. This data is increasingly being hacked these days because hackers' goal is to misuse personal data, which is a profitable financial business for them. Names, dates of birth, health insurance numbers, diagnostic codes, billing information, etc. are among the medical data available to sell for business purposes. Fraudsters use this information to open bank accounts, get passports, or construct forged identification cards in order to manufacture medical equipment or medications. Additionally, according to specialists who have researched cyber-attacks on healthcare institutions, hackers combine a patient number with a bogus provider number, create a file, and then submit a claim with the insurance companies. Since hospitals or clinics have interconnection, easily accessible access points, outdated systems, and a lack of emphasis on cybersecurity for sharing data, hackers may easily obtain enormous amounts of data. Hence, the suggested strategy will be more beneficial for any medical centre or hospital in terms of protecting the privacy of sensitive data, as electronic health records are shared among the physicians, patients, staff, and others, even though other existing approaches show promise. Moreover, future researchers must adhere to this privacy approach in order to safeguard patient confidentiality, cultivate participant confidence, and stop data breaches more appropriately in their further research.

However, it is stated that the study takes into account the analysis through a few objective functions, such as the information hiding failure rate, the information preservation rate, the degree of modification rate, and the fitness function. However, it might potentially function based on decryption time and convergence analysis as well.

## Conclusion

In this work, a privacy preservation technique has been developed in the Enhanced Combined PSO-GWO framework. For this purpose, a sanitized key is utilized, which is yielded from the proposed model. This model introduced a restoration process where the optimal key is applied. Therefore, the most important objective of this study is to introduce a restoration technique where the optimal key is used to recover original information securely by permitted users. Moreover, the performances achieved by the recommended model have been compared with the traditional algorithms and attained the expected outcomes. From the experiments and simulation results, the suggested model was assessed in comparison with the different kind of attacks and the enhanced results were achieved. It is stated that this study applied four types of autism datasets in this regard. For the 24 months autism child dataset, the model showed that it achieved 99.27%, 99.33%, 99.43%, 97.83%, and 93.22% greater than PSO, GA, DE, CSA, as well as AAP-CSA, correspondingly. Similarly, the performance analysis of the recommended framework for 30 months autism child dataset reveals that 99.89%, 99.81%, 99.54%, 99.37% and 99.76% enhancements over PSO, GA, DE, CSA, and AAP-CSA, respectively. Additionally, it performed 90.13%, 11.62%, 86.83%, 11.70% and 96.47% better than PSO, GA, DE, CSA, and AAP-CSA, individually by applying 36 months autism child dataset. Finally, the enhanced framework outperformed as 2.21%, 85.02%, 55.5%, 70.33% and 77.53% to PSO, GA, DE, CSA, and AAP-CSA respectively over the 48 months autism child dataset.

In conclusion, based on the performances presented in the results and discussion section, it is evident that the developed approach surpasses traditional algorithms in terms of handling autism sensitive data or information.

The research will, however, encompass datasets from diverse fields, including banking, social media, and e-commerce, as further studies.

## Data Availability

All autism datasets used in this work were obtained from the Faculty of Education at Universiti Kebangsaan Malaysia. These datasets are not publicly available due to the restrictions of Universiti Kebangsaan Malaysia (an affiliated institution), but are available from the corresponding author on reasonable request for further progress of the research work.

## Code availability

The codes utilized in this work were all sourced from my research work at the Faculty of Information Science and Technology, Universiti Kebangsaan Malaysia. These codes are not publicly available due to the constraints imposed by the Faculty of Information Science and Technology, Universiti Kebangsaan Malaysia (an affiliated institution). However, the corresponding author may provide them upon rational request in order to further the research effort.
